# *Verticillium dahliae* LysM effectors differentially contribute to virulence on plant hosts

**DOI:** 10.1111/mpp.12520

**Published:** 2017-02-14

**Authors:** Anja Kombrink, Hanna Rovenich, Xiaoqian Shi‐Kunne, Eduardo Rojas‐Padilla, Grardy C. M. van den Berg, Emmanouil Domazakis, Ronnie de Jonge, Dirk‐Jan Valkenburg, Andrea Sánchez‐Vallet, Michael F. Seidl, Bart P. H. J. Thomma

**Affiliations:** ^1^ Laboratory of Phytopathology Wageningen University Droevendaalsesteeg 1 Wageningen PB 6708 the Netherlands

**Keywords:** aggressiveness, chitin, comparative genomics, hyphal protection, lineage‐specific effector, tomato, vascular wilt disease

## Abstract

Chitin‐binding lysin motif (LysM) effectors contribute to the virulence of various plant‐pathogenic fungi that are causal agents of foliar diseases. Here, we report the LysM effectors of the soil‐borne fungal vascular wilt pathogen *Verticillium dahliae*. Comparative genomics revealed three core LysM effectors that are conserved in a collection of *V. dahliae* strains. Remarkably, and in contrast with the previously studied LysM effectors of other plant pathogens, no expression of core LysM effectors was monitored *in planta* in a taxonomically diverse panel of host plants. Moreover, targeted deletion of the individual LysM effector genes in *V. dahliae* strain JR2 did not compromise virulence in infections on Arabidopsis, tomato or *Nicotiana benthamiana*. Interestingly, an additional lineage‐specific LysM effector is encoded in the genome of *V. dahliae* strain VdLs17, but not in any other *V. dahliae* strain sequenced to date. Remarkably, this lineage‐specific effector is expressed *in planta* and contributes to the virulence of *V. dahliae* strain VdLs17 on tomato, but not on Arabidopsis or *N. benthamiana*. Functional analysis revealed that this LysM effector binds chitin, is able to suppress chitin‐induced immune responses and protects fungal hyphae against hydrolysis by plant hydrolytic enzymes. Thus, in contrast with the core LysM effectors of *V. dahliae*, this lineage‐specific LysM effector of strain VdLs17 contributes to virulence *in planta*.

## Introduction

To establish infection, fungal plant pathogens secrete effector molecules that manipulate host physiology, including immune responses that are triggered when plant hosts sense invading pathogens (Cook *et al*., 2015; Jones and Dangl, 2006; Rovenich *et al*., 2014). Typically, effectors are small secreted proteins that are species or even lineage‐specific. However, some effectors are more broadly conserved, such as the necrosis‐ and ethylene‐inducing‐like proteins (NLPs) that are produced by bacteria, oomycetes and fungi, and that are particularly known for their phytotoxic activity (Gijzen and Nürnberger, 2006; de Jonge *et al*., 2011). Another group of conserved fungal effectors are the lysin motif (LysM) effectors, which are found in a wide range of fungal species, including plant and animal pathogens, as well as saprophytes (de Jonge and Thomma, 2009; Kombrink and Thomma, 2013).

LysM effectors are defined as secreted proteins that contain no annotated protein domains apart from a varying number of LysM domains, which are carbohydrate‐binding modules that occur in many prokaryotic and eukaryotic proteins (Buist *et al*., 2008; de Jonge and Thomma, 2009). The LysM effectors studied to date belong to various fungal plant‐pathogenic species and have all been found to bind chitin, a homopolymer of unbranched β‐1,4‐linked *N*‐acetylglucosamine (GlcNAc) (de Jonge *et al*., 2010; Marshall *et al*., 2011; Mentlak *et al*., 2012; Takahara *et al*., 2016). Chitin is a major component of fungal cell walls and plays an important role in the interaction between fungal pathogens and their plant hosts (Bowman and Free, 2006; Kombrink *et al*., 2011; Rovenich *et al*., 2016; Sánchez‐Vallet *et al*., 2015). Plants have evolved to recognize chitin as a ‘non‐self’ molecule and to mount an immune response on chitin perception in order to stop fungal infection (Felix *et al*., 1993; Shibuya *et al*., 1993). Several plasma membrane‐localized chitin receptors have been identified in plants that all contain LysM domains (Cao *et al*., 2014; Kaku *et al*., 2006; Miya *et al*., 2007; Wan *et al*., 2008). LysM effectors of various fungal foliar pathogens, namely the tomato leaf mould fungus *Cladosporium fulvum*, the Septoria tritici blotch fungus *Zymoseptoria tritici* (formerly *Mycosphaerella graminicola*), the rice blast fungus *Magnaporthe oryzae* and the Brassicaceae anthracnose fungus *Colletotrichum higginsianum*, have been demonstrated to perturb the activation of chitin‐induced immunity during host colonization and to contribute to virulence (de Jonge *et al*., 2010; Marshall *et al*., 2011; Mentlak *et al*., 2012; Takahara *et al*., 2016). Recently, the crystal structure of the LysM effector Ecp6, secreted by *C. fulvum*, has revealed that Ecp6 suppresses chitin‐triggered immunity through two distinct mechanisms (Sánchez‐Vallet *et al*., 2013). One mechanism involves two LysM domains of a single Ecp6 molecule (LysM1 and LysM3) that cooperatively bind chitin with ultra‐high (pM) affinity and allow Ecp6 to outcompete plant receptors for chitin binding. In addition, the remaining, singular, LysM domain (LysM2) binds chitin and has the capability to suppress chitin‐induced immune responses. However, its relatively low (μM) affinity for chitin probably does not allow this domain to function through the sequestration of chitin fragments, suggesting that LysM2 suppresses chitin‐triggered immunity through another mechanism that potentially involves receptor complex disturbance (Sánchez‐Vallet *et al*., 2013). In addition, the LysM effectors Mg1LysM and Mg3LysM from *Z. tritici* have been found to protect fungal hyphae against degradation by plant chitinases (Marshall *et al*., 2011; Schlumbaum *et al*., 1986). Although the LysM effectors that have been characterized to date are all secreted by foliar pathogens, they are also found in the genomes of soil‐borne pathogens that infect host plants through their roots, such as *Fusarium oxysporum* and *Verticillium dahliae* (de Jonge and Thomma, 2009).

*Verticillium dahliae* is a soil‐borne fungal pathogen that colonizes the xylem vessels of its host plants, resulting in vascular wilt disease (Fradin and Thomma, 2006; Klimes *et al*., 2015; Klosterman *et al*., 2009). *Verticillium dahliae* infects a wide range of dicotyledonous plant species, including economically important crops, such as cotton, lettuce and tomato. Genetic resistance has been characterized in tomato, and it was shown that the cell surface‐localized immune receptor Ve1 confers resistance against strains of *V. dahliae* that belong to race 1 (Fradin *et al*., 2009). However, Ve1 homologues that may recognize *V. dahliae* are widespread in plants (Song *et al*., 2016; Zhang *et al*., 2013). Based on comparative population genomics, it was discovered that Ve1 recognizes the race 1‐specific effector protein Ave1 (for Avirulence on *Ve1* tomato) in order to activate effector‐triggered immunity (de Jonge *et al*., 2012). Furthermore, *Ave1* is required for full virulence on tomato genotypes that lack *Ve1* (de Jonge *et al*., 2012). In addition, comparative population genomics revealed that all *V. dahliae* strains carry lineage‐specific genomic regions that account for 1–5 Mb of their ∼35‐Mb genome and are significantly enriched for *in planta* expressed genes (Faino *et al*., 2015, 2016; de Jonge *et al*., 2013, 2012). Although, in several other plant‐pathogenic species, such as *F. oxysporum* and *Z. tritici*, such lineage‐specific regions are found as small dispensable chromosomes (Goodwin *et al*., 2011; Ma *et al*., 2010), in *V. dahliae* these regions are found as islands within the core chromosomes (Faino *et al*., 2015, 2016; de Jonge *et al*., 2013).

In this article, we characterize the LysM effector gene family of *V. dahliae* which consists of three members in the core genome of *V. dahliae*. Remarkably, one additional LysM effector gene was found in a lineage‐specific region of *V. dahliae* strain VdLs17.

## Results

### Three core LysM effectors are encoded in the *V. dahliae* genome

Initially, LysM effectors were identified in the first available *V. dahliae* genome sequence, that of strain VdLs17 (Klosterman *et al*., 2009). However, as this strain has been isolated from the non‐model plant lettuce and is a rather mild pathogen of tomato and Arabidopsis (K. A. Yadeta and B. P. H. J. Thomma, unpublished data), our research focuses on *V. dahliae* strain JR2 which is particularly aggressive on these plant hosts (de Jonge *et al*., 2013, 2012; Santhanam *et al*., 2013, 2016; Santhanam and Thomma, 2013). Recently, we established a gapless whole‐genome sequence of the *V. dahliae* JR2 strain (Faino *et al*., 2015; http://fungi.ensembl.org/Verticillium_dahliaejr2/Info/Index). The seven LysM effector genes that were initially identified in strain VdLs17 were also found to occur in JR2, except for the lineage‐specific effector VDAG_05180 (de Jonge *et al*., 2013; Klosterman *et al*., 2009). The identification of effector genes is often hampered by erroneous gene annotation (Gibriel *et al*., 2016). Therefore, for the present study, we revisited the corresponding gene models, revealing that not all of the initially identified LysM effector genes were predicted correctly. First, VdLs17 protein VDAG_03096 (VDAG_JR2_Chr8g05800) contains a single LysM domain that only constitutes a minor portion of the total protein (Fig. S1, see Supporting Information). Furthermore, mapping of RNA sequencing reads from samples of *V. dahliae* grown *in vitro* and *in planta* (Faino *et al*., 2012; de Jonge *et al*., 2012) indicated that the initially predicted gene model of the corresponding locus is incorrect, as reads mapped to predicted introns, whereas other parts of the predicted gene, including the LysM domain, were not supported by read mapping (Fig. S1). Thus, VDAG_03096 (VDAG_JR2_Chr8g05800) was disqualified as a bona fide LysM effector. Similarly, deeper analysis of the predicted VdLs17 VDAG_06426 (VDAG_JR2_Chr7g08210) protein sequence by SMART protein domain analysis (http://smart.embl-heidelberg.de) revealed the absence of a signal peptide and the presence of a zinc finger domain (Fig. S1), disqualifying this protein as a bona fide LysM effector. Finally, VdLs17 protein VDAG_08171 (VDAG_JR2_Chr8g01370) was disqualified because of the presence of a glycosylphosphatidylinositol (GPI) anchor, suggesting that it is a membrane‐associated protein, rather than an apoplastic effector protein. The three remaining VdLs17 LysM effectors VDAG_00902, VDAG_04781 and VDAG_06998 are not only encoded by strain JR2 (as VDAG_JR2_Chr1g02480, VDAG_JR2_Chr6g09650 and VDAG_JR2_Chr4g05910, respectively), but also by 18 additional *V. dahliae* strains that were sequenced (Table S1, see Supporting Information) (de Jonge *et al*., 2013, 2012; B. P. H. J. Thomma *et al*., unpublished data), and are further referred to as core VdLysM effectors. As these effectors comprise four, five or six LysM domains, they were named accordingly: Vd4LysM, Vd5LysM and Vd6LysM.

### *VdLysM* sequence analysis in 20 *V. dahliae* strains

**Figure 1:**
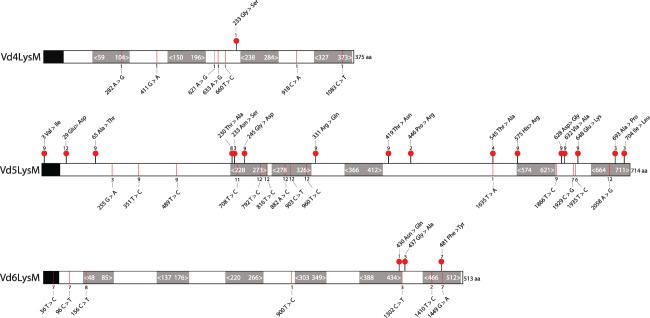
Sequence polymorphisms of core VdLysM effector genes in *Verticillium dahliae* population. The single nucleotide polymorphisms (SNPs) were compared among 20 *V. dahliae* strains. The protein models are based on the annotation of VdLysMs in *V. dahliae* strain JR2. Three core LysM effectors have four to six predicted LysM domains, which are represented by grey squares (amino acid positions are indicated with white font). The N‐terminal black box represents the predicted signal peptide. Sites with synonymous and non‐synonymous nucleotide substitutions are marked by red lines and red dots, respectively. The number above or below the SNP sites indicates the amount of strains that share the same nucleotide polymorphism at that position (detailed information on each individual SNP can be found in Fig. S2).

To investigate sequence polymorphisms in *VdLysM* effector genes within the *V. dahliae* population, we mapped the sequencing reads of 19 *V. dahliae* strains (Table S1) to the genome assembly of strain JR2. Subsequently, we analysed single nucleotide polymorphisms (SNPs) in the different *V. dahliae* strains. This revealed that *Vd4LysM* is the most conserved LysM effector gene, as only one strain (Vd39) showed seven synonymous SNPs and one non‐synonymous SNP (Figs 1, S2, see Supporting Information). *Vd5LysM* displays the most SNPs: 14 synonymous and 16 non‐synonymous SNPs were found involving the majority of the strains. For *Vd6LysM*, seven synonymous and three non‐synonymous SNPs were identified in seven strains in total (Figs 1, S2). Although the sequence variation in *Vd5LysM* exceeds that of the other *VdLysM* effector genes, no evidence for negative or positive selection could be found. No SNPs were observed in LysM effector genes of strains VdLs17, 2009‐605, V152, V52, van Dijk and Vd57 when compared with those of JR2, suggesting that these strains are phylogenetically more closely related to JR2 than the remaining 13 strains.

### Core LysM effectors of *V. dahliae* strain JR2 do not contribute to virulence on tomato

**Figure 2:**
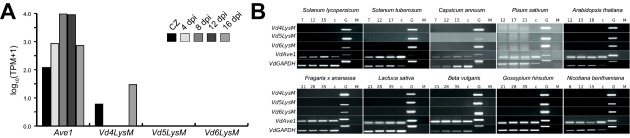
Core VdLysM effector genes of *Verticillium dahliae* strain JR2 are not expressed *in planta*. (A) VdLysM effector gene expression and expression of the *Ave1* effector gene during growth of strain JR2 on Czapek Dox medium (CZ) and during colonization on *Nicotiana benthamiana* at 4, 8, 12 and 16 days post‐inoculation (dpi). (B) *In planta* reverse transcription‐polymerase chain reaction (RT‐PCR) expression analysis of core VdLysM genes. Expression of VdLysM genes could not be detected in 10 different plant species (*Solanum lycopersicum*, *Solanum tuberosum*, *Capsicum annuum*, *Pisum sativum*, *Arabidopsis thaliana*, *Fragaria × ananassa*, *Lactuca sativa*, *Beta vulgaris*, *Gossypium hirsutum* and *N. benthamiana*) at time points between 7 and 35 dpi. Expression was analysed at three time points per species and referenced to the expression of *VdAve1*, and *VdGAPDH* cDNA (c) and genomic DNA (G) from *V. dahliae* cultured *in vitro* were used as controls. Primer pairs were designed to span introns to account for amplification from cDNA or DNA templates. Mock (M) treated plants are also shown.

To test whether the core LysM effectors contribute to the virulence of *V. dahliae* on tomato, gene functional analysis was pursued in *V. dahliae* strain JR2. Previously, RNA sequencing was performed on samples harvested during a time course of *V. dahliae*‐inoculated *Nicotiana benthamiana* plants (Faino *et al*., 2012; de Jonge *et al*., 2012). These data were used to assess the expression of *VdLysM* genes during host invasion. However, no significant *VdLysM* effector gene expression was observed at any of the time points. In contrast, the expression of the functionally analysed *Ave1* effector gene was significantly induced in these samples (de Jonge *et al*., 2013) (Fig. 2A).

Differential *V. dahliae* effector gene induction among host plant species has been observed previously, as the *NLP2* effector gene was found to be induced in tomato and Arabidopsis, but not in *N. benthamiana* (Santhanam *et al*., 2013). Therefore, reverse transcription‐polymerase chain reaction (RT‐PCR) was performed on cDNA generated from samples of tomato (*Solanum lycopersicum*) and Arabidopsis (*Arabidopsis thaliana*) plants inoculated with *V. dahliae* strain JR2 and harvested at different time points. However, again no expression of any of the *VdLysM* effector genes was recorded, whereas expression of *Ave1* as a positive control was clearly detected (Fig. 2B). In a final attempt to detect *in planta* expression of core *VdLysM* effector genes, a taxonomically wide range of plants, including potato (*Solanum tuberosum*), pepper (*Capsicum annuum*), Australian tobacco (*N. benthamiana*), pea (*Pisum sativum*), strawberry (*Fragaria × ananassa*), lettuce (*Lactuca sativa*), beet (*Beta vulgaris*) and cotton (*Gossypium hirsutum*), were inoculated with *V. dahliae* strain JR2 and *VdLysM* effector gene expression was monitored. However, expression of *VdLysM* effector genes could not be detected in any of these plant species, whereas the expression of the *Ave1* effector gene was observed in all hosts (Fig. 2B).

**Figure 3:**
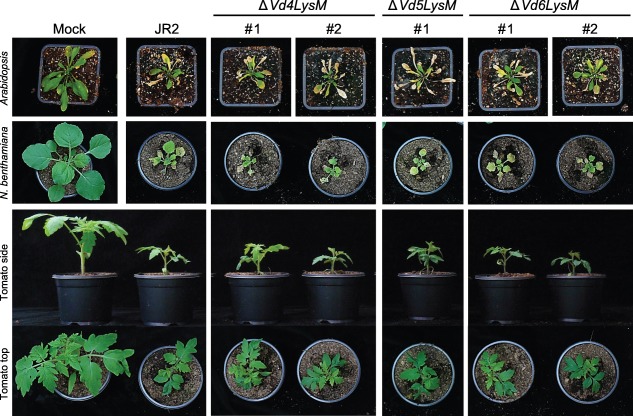
Core VdLysM effector genes do not contribute to the virulence of *Verticillium dahliae* strain JR2. Infection assay of wild‐type and VdLysM effector gene deletion strains on Arabidopsis, *Nicotiana benthamiana* and tomato plants showing two deletion strains for each VdLysM effector gene, with the exception of *Vd5LysM*. At 14 (tomato, *N. benthamiana*) or 21 days post‐inoculation (Arabidopsis), photographs were taken of one representative plant out of six that were inoculated with the same *V. dahliae* genotype. The infection assays on Arabidopsis, *N. benthamiana* and tomato were repeated three times with similar results.

As it cannot be excluded that *VdLysM* effector gene expression is induced at low levels only, or only at specific time points or at particular sites, further investigations into the possible contribution of the core *VdLysM* effector genes to the virulence of *V. dahliae* strain JR2 were evaluated by targeted deletion. Multiple deletion strains were obtained for the LysM effector genes *Vd4LysM*, *Vd5LysM* and *Vd6LysM*. Importantly, none of the deletion strains obtained showed any phenotypic deviations from the wild‐type JR2 strain on cultivation *in vitro* (data not shown). Subsequent virulence assays revealed that Arabidopsis, *N. benthamiana* and tomato plants inoculated with the wild‐type strain or with deletion strains for *Vd4LysM*, *Vd5LysM* and *Vd6LysM* showed similar disease development; the timing and degree of stunting of the plants and wilting of the leaves were similar on inoculation with the various genotypes (Fig. 3). Thus, the lack of *VdLysM* effector gene expression, together with the observation that *VdLysM* deletion strains are not affected in virulence on Arabidopsis, *N. benthamiana* and tomato, strongly suggest that the core VdLysM effectors do not play an important role during host colonization.

### A lineage‐specific LysM effector is required for full virulence on tomato, but not on *N. benthamiana* or Arabidopsis

It has been demonstrated previously that lineage‐specific effectors of *V. dahliae* strain JR2 contribute significantly to virulence on tomato (de Jonge *et al*., 2013), suggesting that lineage‐specific regions of individual *V. dahliae* genotypes are important for the development of aggressiveness on particular host plants. Intriguingly, one LysM effector gene, *VDAG_05180*, was originally identified in strain VdLs17, but could not be found in strain JR2 or in any of the other 18 additional *V. dahliae* strains that were sequenced, revealing that *VDAG_05180* is a lineage‐specific effector gene. Interestingly, apart from VdLs17, none of the other sequenced *V. dahliae* strains carries lineage‐specific LysM effector genes.

**Figure 4:**
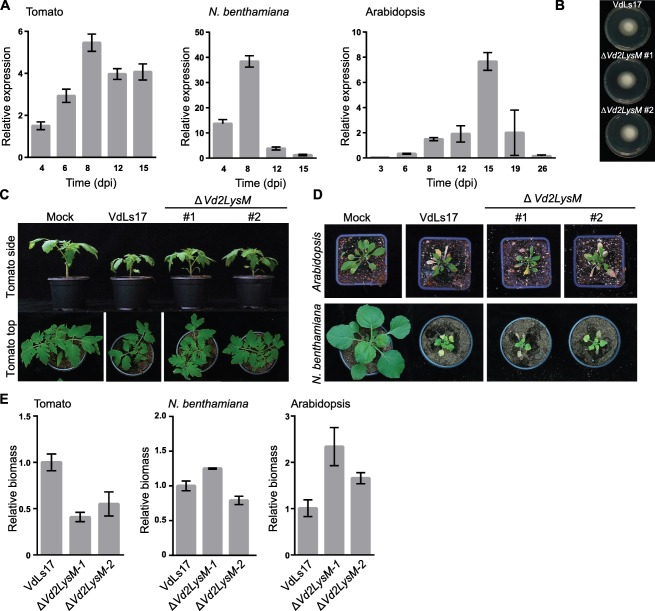
Lineage‐specific LysM effector Vd2LysM contributes to the virulence of *Verticillium dahliae* strain VdLs17 on tomato. (A) *Vd2LysM* expression during colonization of *V. dahliae* strain VdLs17 on tomato, *N. benthamiana* and Arabidopsis plants at 2–26 days post‐inoculation (dpi). (B) Morphology of wild‐type *V. dahliae* strain VdLs17 and two *Vd2LysM* deletion strains after 7 days of incubation on potato dextrose agar (PDA) medium at room temperature. (C) Photographs of representative tomato plants out of eight plants that were either mock‐inoculated or inoculated with wild‐type *V. dahliae* strain VdLs17 or two deletion strains of *Vd2LysM* at 14 dpi. The infection assay was repeated three times with similar results. (D) Photographs of representative *N. benthamiana* and Arabidopsis plants out of eight plants that were either mock‐inoculated or inoculated with wild‐type *V. dahliae* strain VdLs17 or two deletion strains of *Vd2LysM* at 14 dpi (*N. benthamiana*) and 21 dpi (Arabidopsis). The assay was repeated three times with similar results. (E) Fungal biomass accumulation in tomato, *N. benthamiana* and Arabidopsis plants inoculated with wild‐type strain VdLs17 or Vd2LysM deletion strains at 14 dpi (tomato, *N. benthamiana*) and 21 dpi (Arabidopsis). Error bars represent the standard error of three replicate experiments.

It has been shown that the VdLs17 lineage‐specific LysM effector which contains two LysM domains, and is hence designated Vd2LysM, contributes to virulence on tomato (de Jonge *et al*., 2013). Here, the expression of *Vd2LysM* during infection of *V. dahliae* strain VdLs17 on tomato, *N. benthamiana* and Arabidopsis was investigated using real‐time PCR, and expression was detected at each of the time points monitored (Fig. 4A). To test whether Vd2LysM contributes to virulence, *Vd2LysM* deletion strains were inoculated on these host plants. Importantly, none of the transformants showed morphological anomalies on growth *in vitro* when compared with the VdLs17 wild‐type strain (Fig. 4B). As noted previously (de Jonge *et al*., 2013), the *Vd2LysM* deletion strains showed significantly reduced virulence on inoculation on tomato when compared with the wild‐type strain VdLs17 (Fig. 4C). The tomato plants that were inoculated with the wild‐type strain showed stronger stunting than plants that were inoculated with two independent *ΔVd2LysM* strains (Fig. 4C). In accordance with the reduced symptom development, real‐time PCR quantification of the fungal biomass showed that the *ΔVd2LysM* strains produced significantly less biomass than the wild‐type (Fig. 4E). These results show that the lineage‐specific LysM effector gene *Vd2LysM* plays a role in the virulence of *V. dahliae*. To investigate whether the contribution to virulence extends to other plant hosts as well, the *Vd2LysM* deletion strains were inoculated onto *N. benthamiana* and Arabidopsis plants. Intriguingly, the virulence of the *Vd2LysM* deletion strains appeared to be comparable with that of the wild‐type strain on these plant species, as all genotypes induced similar symptomatology on the inoculated plants and real‐time PCR measurements of fungal biomass did not reveal compromised fungal host colonization (Fig. 4D, E). On Arabidopsis, it seems that *Vd2LysM* deletion leads to enhanced colonization.

### *Vd2LysM* binds chitin

**Figure 5:**
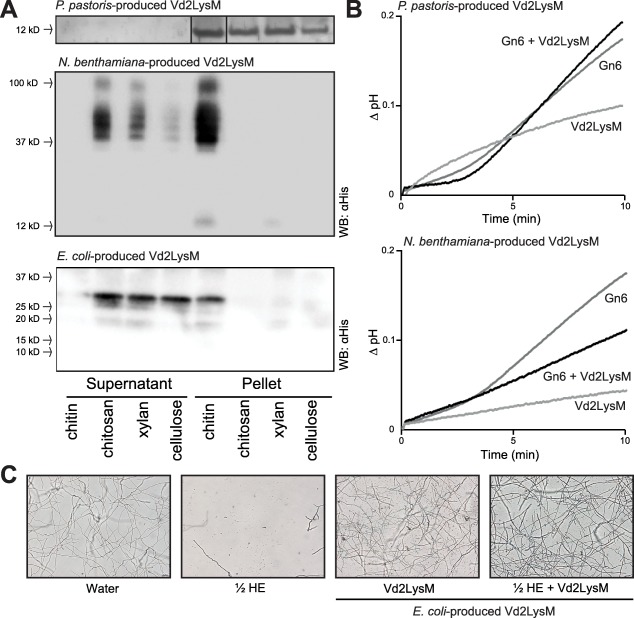
Vd2LysM is a chitin‐binding protein that suppresses chitin‐induced immune responses. (A) Vd2LysM produced in *Pichia pastoris*, *Nicotiana benthamiana* and *Escherichia coli* was used in affinity precipitation experiments with the insoluble polysaccharides chitin, chitosan, xylan and cellulose. After sodium dodecylsulfate‐polyacrylamide gel electrophoresis (SDS‐PAGE) and Coomassie brilliant blue staining for *P. pastoris*‐produced Vd2LysM or western blot analysis for *in planta*‐ and *Escherichia coli*‐produced Vd2LysM, the proteins are observed in the insoluble pellet fraction or in the supernatant fraction. Vd2LysM produced in *P. pastoris* is observed in the insoluble pellet fraction of all polysaccharides. Vd2LysM produced *in planta* and in *E. coli* precipitates with chitin, but not with chitosan, xylan or cellulose. (B) pH shift measurements in a tomato cell suspension after the addition of 1 nm chitin or 1 nm chitin that was pre‐incubated with 10 nm Vd2LysM produced in either *P. pastoris* or *in planta*. The graphs show a representative experiment out of two to four experiments with similar results. (C) Micrographs of *Fusarium oxysporum* f.sp. *lycopersicum* strain 4287 taken 23 h after the addition of water, hydrolytic enzyme mix (HE) or Vd2LysM and pre‐treated with Vd2LysM followed by treatment with hydrolytic enzyme mix. The figure is representative of three independent experiments.

Previously characterized LysM effectors of fungal plant pathogens have been demonstrated to bind chitin (de Jonge *et al*., 2010; Marshall *et al*., 2011; Mentlak *et al*., 2012; Takahara *et al*., 2016). Therefore, the chitin‐binding ability of Vd2LysM was tested. To this end, Vd2LysM was heterologously produced in the yeast *Pichia pastoris*, which has been used previously for the production of LysM effectors from other fungi (Kombrink, 2012). Subsequently, the purified Vd2LysM protein was used in affinity precipitation assays with the insoluble carbohydrates chitin, chitosan, xylan and cellulose. We observed that Vd2LysM precipitated with all carbohydrates tested (Fig. 5A), which suggests that the protein precipitates by itself rather than binds to any of these carbohydrates. To investigate this further, Vd2LysM was subjected to glycan‐array analysis to test the binding affinity for approximately 600 glycans, taking *C. fulvum* Ecp6 as a control. As demonstrated previously, Ecp6 specifically binds to the chitin oligosaccharides (GlcNAc)_3_, (GlcNAc)_5_ and (GlcNAc)_6_ that are present on the array (de Jonge *et al*., 2010). In contrast, Vd2LysM does not bind to any of the glycans on the array (Fig. S3, see Supporting Information). Based on these findings, we conclude that *P. pastoris‐*produced Vd2LysM probably precipitates spontaneously in the affinity precipitation assay.

Recently, a crystal structure has been obtained of *C. fulvum* Ecp6, which was heterologously produced in *P. pastoris*. The crystal structure of the protein contained chitin, arguably derived from *P. pastoris* during protein production, in a composite‐binding groove of two LysM domains (Sánchez‐Vallet *et al*., 2013). To exclude the possibility that potential Vd2LysM substrate‐binding sites were occupied by *P. pastoris* chitin, the effector was produced in *N. benthamiana*. The purified protein was subsequently tested for chitin‐binding ability in an affinity precipitation assay using chitin, chitosan, xylan and cellulose. Interestingly, *N. benthamiana*‐produced Vd2LysM bound to chitin, but not to any of the other carbohydrates (Fig. 5A). Furthermore, we observed that *N. benthamiana‐*produced Vd2LysM migrated as multiple bands on the gel, which were not visible with *P. pastoris‐*produced Vd2LysM, which migrated as a single band of the expected 17 kDa. This may indicate that Vd2LysM produced in *N. benthamiana* forms oligomers. Collectively, these findings suggest that Vd2LysM is a chitin‐binding effector protein, and the chitin that is readily available during heterologous production in *P. pastoris* occupies the Vd2LysM substrate‐binding sites.

### Vd2LysM suppresses chitin‐induced immune responses and protects hyphae against degradation by plant hydrolytic enzymes

Previously, LysM effectors from various fungal plant pathogens have been demonstrated to suppress the chitin‐induced pH shift in a tomato cell suspension, which is indicative of the ability of the effector to perturb chitin‐induced host immune responses (Felix *et al*., 1993; de Jonge *et al*., 2010; Marshall *et al*., 2011; Mentlak *et al*., 2012; Takahara *et al*., 2016). Both *in planta‐*produced and *P. pastoris*‐produced Vd2LysM were tested for this capacity, as described previously (de Jonge *et al*., 2010). Interestingly, *P. pastoris*‐produced Vd2LysM, which is not able to bind chitin, did not suppress the chitin‐induced pH shift, whereas *N. benthamiana*‐produced Vd2LysM, which is able to bind chitin, suppressed the chitin‐induced immune response (Fig. 5B). These results suggest that Vd2LysM is able to suppress chitin‐triggered immune responses during *V. dahliae* colonization of tomato.

As the *Z. tritici* LysM effectors Mg3LysM and Mg1LysM have been demonstrated previously to protect fungal hyphae against degradation by plant hydrolytic enzymes (Marshall *et al*., 2011), we also intended to test Vd2LysM for this activity. However, the yield of *in planta*‐produced Vd2LysM was too low to perform such assays. Therefore, we pursued Vd2LysM production in yet another heterologous system: *Escherichia coli*. Similar to *N. benthamiana*‐produced Vd2LysM, *E. coli*‐produced Vd2LysM was found to bind chitin, but no other insoluble carbohydrates tested (Fig. 5A). Moreover, *E. coli*‐produced Vd2LysM also showed signs of oligomerization. Interestingly, similar to the *Z. tritici* LysM effectors Mg3LysM and Mg1LysM, Vd2LysM was also found to protect fungal hyphae against degradation by plant hydrolytic enzymes (Fig. 5C).

### LysM domains of Vd2LysM are more similar to those of previously characterized LysM effectors than to those of core VdLysM effectors

**Figure 6:**
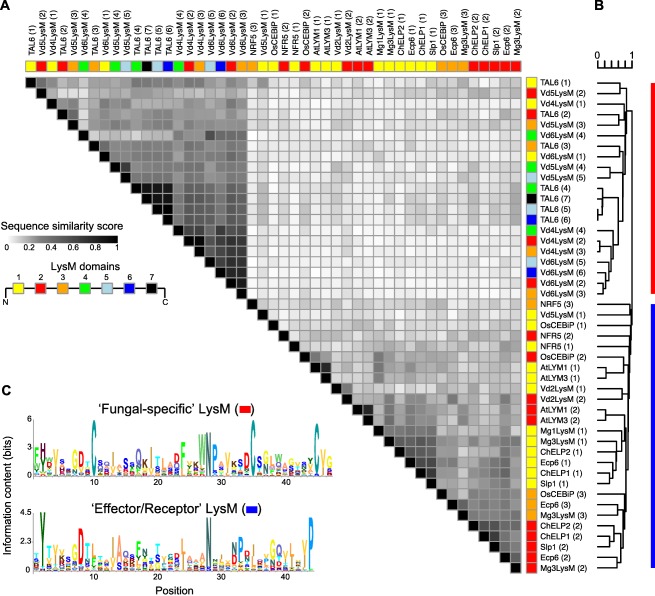
Sequence similarity between LysM domains. (A) Pairwise comparisons of the LysM domains of fungal and plant LysM proteins. Normalized sequence similarities between LysM domains are displayed in the heat map. The colour annotation above the heat map indicates the relative position of each LysM domain within the LysM protein. (B) Hierarchical clustering (UPGMA clustering) based on the (dis)‐similarity matrix of all pairwise LysM domain comparisons is shown above and on the side, and the clustering is used to order the rows and columns of the heat map. (C) Sequence logos of LysM domains identified in fungal effectors as well as in plant receptors (blue) and LysM domains that have been defined previously as ‘fungal specific’.

Vd2LysM is presently the only LysM effector of *V. dahliae* for which a role in virulence could be shown, and which is able to perturb chitin‐centred host immune responses, as described previously for Ecp6, Mg1LysM, Mg3LysM, Slp1, ChELP1 and ChELP2 (de Jonge *et al*., 2010; Marshall *et al*., 2011; Mentlak *et al*., 2012; Takahara *et al*., 2016). Remarkably, the LysM domains of Vd2LysM are more similar to those of Ecp6, Mg1LysM, Mg3LysM, Slp1, ChELP1 and ChELP2 than to those of the core VdLysM effectors (Fig. 6). To further investigate this, we carried out a comparative LysM domain analysis with functionally characterized plant and fungal proteins (Fig. 6). The fungal LysM domains fall into two separate clades (Fig. 6B). One clade was nearly exclusively formed by LysMs of plant receptors and LysMs of fungal effectors with a role in virulence, including Vd2LysM (Bolton *et al*., 2008; Marshall *et al*., 2011; Mentlak *et al*., 2012; Takahara *et al*., 2016). This suggests that fungi and plants produce LysM proteins with a conserved motif, and thus possibly originate from a common ancestor. Interestingly, the second clade solely contains the LysMs of the *V. dahliae* core effectors and the *Trichoderma atroviride* LysM effector TAL6, which has been shown previously to specifically inhibit the germination of conidia of *Trichoderma* spp. and has been proposed to act in fungal development rather than in host interactions (Seidl‐Seiboth *et al*., 2013) (Fig. 6B). Intriguingly, the two clades are characterized by highly divergent consensus motifs for their LysMs, with the ‘fungal‐specific’ LysMs having three highly conserved cysteine residues that are completely lacking from the ‘effector/receptor’ LysMs. Collectively, these findings may suggest that the core LysM effectors of *V. dahliae*, in contrast with Vd2LysM, do not act in host interactions, but possibly in other physiological processes.

## Discussion

Here, we have described the characterization of the LysM effector catalogue of the soil‐borne vascular wilt pathogen *V. dahliae*. In‐depth analysis revealed that three *VdLysM* effector genes occur in the core genome of all strains for which a genome sequence is available. In addition, a lineage‐specific *VdLysM* effector gene, *Vd2LysM*, occurs in strain VdLs17 and not in any of the other strains that have been sequenced to date. We initially reasoned that the ubiquitous occurrence of the three core LysM effector genes suggests that these are required for host colonization by *V. dahliae*. However, a role in virulence cannot be attributed to any of these core LysM effectors. Intriguingly, the lineage‐specific Vd2LysM effector was found to contribute to host‐specific virulence of strain VdLs17.

LysM effectors are found in fungal species with various lifestyles, but, thus far, have only been characterized as virulence factors of plant‐pathogenic fungi (de Jonge *et al*., 2010; Marshall *et al*., 2011; Mentlak *et al*., 2012; Takahara *et al*., 2016). Moreover, they have only been studied in foliar pathogens with a (relatively) narrow host range: *C. fulvum* (Ecp6), *Z. tritici* (Mg1LysM and Mg3LysM), *M. oryzae* (Slp1) and *C. higginsianum* (ChELP1 and ChELP2) (de Jonge *et al*., 2010; Marshall *et al*., 2011; Mentlak *et al*., 2012; Takahara *et al*., 2016). In contrast, *V. dahliae* is a soil‐borne vascular plant pathogen that infects a broad range of host plants. The expression of the previously characterized LysM effectors is induced during the early stages of infection of the respective pathogens, when evasion of recognition by the host is of particular importance to facilitate tissue colonization. The expression of *Vd2LysM* peaks at about 1 week after inoculation, which is when *V. dahliae* biomass is accumulating in the xylem and before wilting and necrosis of the host tissue are visible (Fig. 4A) (Fradin and Thomma, 2006). Ecp6, Mg3LysM, Slp1, ChELP1 and ChELP2 have been demonstrated to function as suppressors of chitin‐induced host immunity and, similar to these proteins, Vd2LysM suppresses chitin‐induced medium alkalinization in a tomato cell suspension. This strongly suggests that Vd2LysM contributes to the virulence of *V. dahliae* through the perturbation of the activation of chitin‐triggered host immunity. However, in contrast with Ecp6, Slp1, ChELP1 and ChELP2, and similar to Mg3LysM, Vd2LysM is also able to protect fungal hyphae against degradation by plant hydrolytic enzymes. Thus, Vd2LysM plays a broad role in overcoming chitin‐centred host immune responses (Sánchez‐Vallet *et al*., 2015).

Considering the widespread occurrence of chitin receptor homologues in plant species, and the observation that LysM effectors of diverse plant pathogens have the ability to suppress chitin‐triggered immunity, it seems that this ability is fundamental for fungal plant pathogens to establish infections on their hosts. As most of the sequenced *V. dahliae* strains are pathogenic on tomato, these strains probably employ molecules other than Vd2LysM to overcome chitin‐centred host immune responses (Sánchez‐Vallet *et al*., 2015). Alternatively, *V. dahliae* may employ other mechanisms to prevent the activation of chitin‐triggered immune responses (Rovenich *et al*., 2016; Sánchez‐Vallet *et al*., 2015). For example, the α‐1,3‐glucan synthase gene has been demonstrated to be important for the virulence of several fungal pathogens, as the α‐1,3‐glucan layer around hyphae reduces the accessibility of fungal chitin to host chitinases and, consequently, prevents the release of free chitin fragments (Fujikawa *et al*., 2012). In addition, fungal species have been found to secrete chitin deacetylases that convert chitin into chitosan, which is a poor inducer of immune responses (Gough and Cullimore, 2011).

Structural analysis of the *C. fulvum* LysM effector Ecp6 revealed that the first and third LysM domains cooperatively bind one chitin molecule with ultra‐high (pM) affinity through intramolecular LysM dimerization, whereas the second, singular LysM domain displays low micromolar affinity (Sánchez‐Vallet *et al*., 2013). Nevertheless, both binding sites are able to suppress chitin‐triggered immune responses (Sánchez‐Vallet *et al*., 2013). Intriguingly, all characterized LysM effectors that have the ability to suppress chitin‐triggered immune responses either have three (Ecp6, Mg3LysM) or two (Slp1, ChELP1, ChELP2, Vd2LysM) LysM domains. It remains unknown whether the LysM effectors with two LysM domains contain one composite chitin‐binding site that is composed of the coordinated action of two LysM domains, or two binding sites of separate, singular LysM domain activities. Consequently, the mechanism by which Vd2LysM perturbs the activation of chitin‐triggered immunity requires further investigation.

Recently, evolutionary analyses and consensus pattern profiling of fungal LysM domains have led to their classification into two groups (Akcapinar *et al*., 2015). The clustering into two phylogenetic clades across taxonomic boundaries is consistent with the presence of a characteristic cysteine residue pattern present in 80% of fungal‐specific LysMs (Akcapinar *et al*., 2015) (Fig. 6C). Remarkably, the group of fungal LysMs which do not display the conserved cysteine residues contains all of the LysM domains of previously characterized LysM effectors from fungal pathogens (Bolton *et al*., 2008; Marshall *et al*., 2011; Mentlak *et al*., 2012; Takahara *et al*., 2016) (Fig. 6C). It has been noted previously that LysM domains from saprotrophs, mutualists and mycoparasitic fungi are under‐represented in this clade (Akcapinar *et al*., 2015), pointing to the significantly diverging roles of LysM proteins. To predict the putative roles of the core LysM effectors of *Verticillium*, we carried out a comparative LysM domain analysis with functionally characterized plant and fungal proteins (Fig. 6). Consistent with previous findings (Akcapinar *et al*., 2015), the fungal LysM domains analysed here largely fall into two separate clades (Fig. 6B). Interestingly, the *V. dahliae* core effectors and TAL6 from the mycoparasitic fungus *T. atroviride*, which selectively inhibits the spore germination of *Trichoderma* spp. (Seidl‐Seiboth *et al*., 2013), grouped into the same clade (Akcapinar *et al*., 2015) (Fig. 6B). Considering the similarity of the core LysM effectors of *V. dahliae* to TAL6, we hypothesize that they could function in other physiological processes rather than virulence. Although the (single gene) mutants do not display phenotypic deviations from the wild‐type strain, further experimental support is required to confirm this hypothesis. Originally, the LysM domain was identified in bacterial lysozymes that bind and hydrolyse peptidoglycan components of bacterial cell walls (Buist *et al*., 2008). Therefore, it is possible that fungal LysMs also display peptidoglycan‐specific binding activities, which could help fungi to outcompete bacterial competitors (Kombrink and Thomma, 2013). Similar to the LysM effector functions of plant‐colonizing fungi, LysM effectors of saprotrophs could protect fungal hyphae from hydrolytic enzyme attack secreted by mycoparasites.

In conclusion, we have demonstrated that a lineage‐specific LysM effector of *V. dahliae* contributes to virulence, whereas three LysM effectors that are present in the core genome do not seem to play a role during host colonization. This finding confirms previous observations that, in particular, those effectors that are encoded in lineage‐specific regions of *V. dahliae* genomes are important for fungal aggressiveness (de Jonge *et al*., 2013). It is tempting to speculate that the core LysM effectors play other roles in fungal life. Therefore, it might be worthwhile to investigate whether the core LysM effectors contribute to fungal growth in other stages of the *V. dahliae* life cycle that were not covered in this study, for example, during saprophytic growth or during survival as resting structures in the soil.

## Experimental Procedures

### VdLysM effector gene expression analysis

Conidiospores of *V. dahliae* strain JR2 were inoculated on potato dextrose agar (PDA) plates and grown at room temperature for 7 days before harvest and dilution in 40 mL of potato dextrose broth (PDB) to a concentration of 1 × 10^6^ conidia/mL that was used as inoculum for plant inoculation. Plants of *S. lycopersicum*, *S. tuberosum*, *C. annuum*, *P. sativum*, *A. thaliana*, *F. × ananassa*, *L. sativa*, *B. vulgaris*, *G. hirsutum* and *N. benthamiana* were grown in the glasshouse at 21°C/19°C during 16‐h/8‐h light/dark photoperiods, respectively, with a relative humidity of ∼75% and 100 W/m^2^ supplemental light when the light intensity dropped below 150 W/m^2^. At 10 days post‐germination, the plants were inoculated with *V. dahliae* or PDB as mock treatment by dipping of the roots of uprooted plants for 6 min before transfer to fresh soil. Three time points were set for each species and five inoculated plants were collected per time point.

A two‐step RT‐PCR was performed to test the expression of VdLysM effectors. RNA was extracted from all sampled plant material using the QuickRNA™ MiniPrep kit (Zymo Research, Irvine, CA, USA) following the protocol provided by the manufacturer. cDNA was synthesized from 1 μg of RNA from each sample by M‐MMLV reverse transcriptase (Promega, Madison, WI, USA) according to the manufacturer's instructions. cDNA and genomic DNA from *V. dahliae* strain JR2 grown *in vitro* on PDB were used as controls. Expression analysis was performed by PCR using 2 μL of cDNA as template with the following protocol: 5 min at 95°C, followed by 32 cycles of 95°C for 30 s, 58°C for 30 s and 72°C for 1 min. For the strawberry, lettuce, beet and cotton samples, a second PCR was needed to detect the expression of the control genes, owing to the low levels of *V. dahliae* biomass in infected plants. This second PCR was performed by taking 2 μL of the first PCR as template and run with the same temperature profile, but for only 15 cycles. The primers listed in Table S2 (see Supporting Information) were used to amplify transcripts from *Vd4LysM*, *Vd5LysM* and *Vd6LysM*. *VdAVe1* and *VdGAPDH* were used as controls for effector induction *in planta* and presence of *V. dahliae* in the sample, respectively. The primers were designed to span introns to discriminate between amplicons derived from cDNA or DNA templates as a control for DNA contaminations in the synthesized cDNA. Products from all samples were visualized by gel electrophoresis in a 2% agarose gel.

### Functional analysis of *VdLysM* effector genes

*VdLysM* effector gene deletion strains were generated by amplification of flanking sequences of the coding sequences using the primer sets listed in Table S2. PCR products were subsequently cloned into pRF‐HU2 (Frandsen *et al*., 2008). *Verticillium dahliae* transformation and subsequent inoculations on tomato (cv. Motelle and MoneyMaker) plants to assess the virulence of the knock‐out mutants were performed as described by Fradin *et al*. (2009). In one experiment, six to eight plants were used per inoculation with wild‐type or deletion strains, and the experiment was repeated at least three times. Plants were regularly inspected and representative plants were photographed at 12 and 21 days post‐inoculation (dpi). For biomass quantification, the roots and stem below the cotyledons of four plants per *V. dahliae* genotype were flash frozen in liquid nitrogen. The samples were ground to powder, and an aliquot was used for DNA isolation (Fulton *et al*., 1995). Real‐time PCR was conducted with primer sets SlRub‐F1/SlRub‐F2 for tomato *RuBisCo* and VdGAPDH‐F/VdGAPDH‐R for *V. dahliae GAPDH* (Table S2). For expression analyses, 3‐week‐old *N. benthamiana* plants were inoculated with strain VdLs17 as described previously (Fradin *et al*., 2009), harvested at 4, 6, 8 and 12 dpi, and flash frozen in liquid nitrogen. Total RNA was extracted using the RNeasy Kit (Qiagen, Hilden, Germany) and cDNA was synthesized by SuperScript III (Invitrogen, Carlsbad, CA, USA). Real‐time PCR was conducted with primer sets Q‐VdGAPDH‐F/Q‐VdGAPDH‐R for *V. dahliae GAPDH* and Q‐Vd2LysM‐F/Q‐Vd2LysM‐R for *V. dahliae Vd2LysM* (Table S2).

### *VdLysM* sequence analysis and screening of lineage‐specific regions for LysM effectors

The sequencing reads of 19 *V. dahliae* stains (Table S1) were mapped to the genome assembly of strain JR2 using BWA with default settings (Li and Durbin, 2009). Subsequently, SNPs in genes encoding core LysM proteins were detected using FreeBayes with default settings (Garrison and Marth, 2012). The effects of SNPs were predicted using VEP (McLaren *et al*., 2010).

We performed pairwise whole‐genome alignments of all *V. dahliae* strains with NUCmer (using maxmatch option and alignments were filtered by 90% identity), which is part of the mummer package (Kurtz *et al*., 2004). Subsequently, for each reference strain, the aligned regions were extracted using bedtools (Quinlan and Hall, 2010). Regions in the reference strain that are present in less than 18 strains were considered as lineage‐specific regions. Subsequently, we used all the LysM protein sequences that were compared in this study as queries for a blast search (tblastn; Altschul *et al*., 1990) against the lineage‐specific regions.

### Heterologous Vd2LysM expression in *P. pastoris*

*Vd2LysM* was cloned into *P. pastoris* expression vector pPic9 (Invitrogen) after performing PCR using primers to add the N‐terminal HIS‐ and FLAG‐tag and *Eco*RI and *Not*I restriction sites for directional cloning (Table S1). Subsequently, *P. pastoris* strain GS115 was transformed and a selected clone was cultured in a fermentor (Bioflo 3000) as described previously (Rooney *et al*., 2005). After removal of cells and concentration of the culture medium, the HIS‐tagged protein was purified using an Ni‐NTA column (Qiagen) according to the manufacturer's protocol. The final protein concentration was determined spectrophotometrically at 280 nm and confirmed using the Pierce BCA Protein Assay Kit (Thermo Scientific, Waltham, MA, USA) with bovine serum albumin (BSA) as a standard.

### *In planta* production of Vd2LysM

PCR was performed to add *Nhe*I and *Sac*I restriction sites to *Vd2LysM*. Using directional cloning, *Vd2LysM* was cloned into vector pHYG, a modified version of the expression vector pMDC32 (Curtis and Grossniklaus, 2003). The expression vector was subsequently transferred into *Agrobacterium tumefaciens* strain MOG101. Infiltration of 4–5‐week‐old *N. benthamiana* with *Agrobacterium tumefaciens* was performed as described by Westerhof *et al*. (2012). Four days after infiltration, leaves were harvested and flash frozen in liquid nitrogen. Plant material was ground and homogenized in ice‐cold extraction buffer [1% v/v Tween‐20, 2% w/v immobilized polyvinylpolypyrrolidone (PVPP), 300 mm NaCl, 50 mm NaH_2_PO_4_, 10 mm imidazole, 1 mm dithiothreitol (DTT), pH 7.4] using 2 mL/g fresh plant material. After 30 min of homogenization at 4°C, the crude extract was centrifuged at 16 000 ***g*** at 4°C. The supernatant was further cleaned using a miracloth filter, and an Ni‐NTA Superflow column (Qiagen) was used to purify the HIS‐tagged protein, according to the manufacturer's protocol. The final protein concentration was determined spectrophotometrically at 280 nm and confirmed using the Pierce BCA Protein Assay Kit (Thermo Scientific) with BSA as a standard.

### *Escherichia coli* production of Vd2LysM

The coding sequence of Vd2LysM was amplified from *V. dahliae* cDNA and cloned into the pETSUMO (Invitrogen) expression vector according to the manufacturer's instructions prior to *E. coli* Origami (DE3) transformation. A single transformant was selected and grown in Luria broth medium until an optical density at 600 nm (OD_600_) of 0.9 was reached. Heterologous production of Vd2LysM was induced with 1 mm Isopropyl β‐D‐1‐thiogalactopyranoside (IPTG) at 26°C during ∼20 h. Cell pellets were lysed using lysozyme from chicken egg (Sigma, St Louis, MO, USA) and Vd2LysM was purified from the soluble protein fraction using an Ni^2+^‐NTA Superflow column (Qiagen). Purified protein was dialysed against 200 mm NaCl and concentrated over Amicon ultracentrifugal filter units (Molecular weight cut‐off (MWCO) = 3 kDa; Millipore, Billerica, MA, USA).

### Sequence similarity between LysM domains

LysM domains within fungal and plant proteins were identified using the SMART database (including PFAM domain and outlier detection) (Schultz *et al*., 1998). Sequence similarities between LysM domains were approximated using the Needleman–Wunsch algorithm, which has been implemented in the EMBOSS package (Rice *et al*., 2000). The Needleman–Wunsch score was further normalized to the range 0–1 by NW_*ij*_norm = NW_*ij*_/max(NW_*ii*_, NW_*jj*_). Normalized Needleman–Wunsch scores were displayed in R. Moreover, hierarchical clustering unweighted pair group method with arithmetic mean (UPGMA) based on the (dis)‐similarity matrix of all pairwise LysM domains was performed in R. Sequence logos for effector/receptor LysM domains and other fungal LysM domains were created with Skylign (Wheeler *et al*., 2014). LysM domain sequences were aligned using mafft (global setting) (Katoh *et al*., 2002).

### Affinity precipitation assays

These assays were performed as described by van den Burg *et al*. (2016) with 100 μg/mL Vd2LysM. The protein was incubated at room temperature for 1 h with 3 mg of insoluble polysaccharides with gentle rocking.

## Supporting information

Additional Supporting Information may be found in the online version of this article at the publisher's website.

**Fig. S1** Disqualification of two previously identified *Verticillium dahliae* LysM effector genes. Two originally identified VdLysM effector genes are not predicted correctly. The initially predicted gene model of LysM effector gene *VDAG_03096* is not supported by the mapping of RNA sequencing reads (in red). Reads map to predicted introns (in yellow), whereas some coding parts of the gene (in green), including the LysM domain that constitutes only a small part of the predicted protein, is not supported by reads. SMART prediction using the amino acid sequence encoded by VDAG_06426 reveals the absence of a signal peptide and the presence of a zinc finger domain. Also in this case, the LysM domain constitutes only a small portion of the predicted protein.

**Fig. S2** Sequence polymorphisms of core VdLysM effectors in the *Verticillium dahliae* population. Synonymous and non‐synonymous polymorphisms and their occurrence in 19 *V. dahliae* strains are indicated per VdLysM effector gene, and nucleotide and amino acid positions of polymorphisms are indicated, respectively.

**Fig. S3** Glycan array analysis of *Pichia pastoris*‐produced Vd2LysM and Ecp6. Relative fluorescence (RFU, relative fluorescence unit) on scanning of a glycan array that contains probes for 406 glycans after hybridization with Vd2LysM and with Ecp6 as a control. Only Ecp6 hybridizes to the array, and only to 170–172, representing (GlcNAc)_6_, (GlcNAc)_5_ and (GlcNAc)_3_, respectively.

**Table S1** *Verticillium dahliae* strains used in this study.

**Table S2** Primers used in this study.
