# Supplementary figures and images for: Verticillium dahliae LysM effectors differentially contribute to virulence on plant hosts
Source: Mol Plant Pathol. 2017 Feb 14;18(4):596–608. doi: 10.1111/mpp.12520 (PMC6638240; doi:10.1111/mpp.12520)

VDAG\_03096

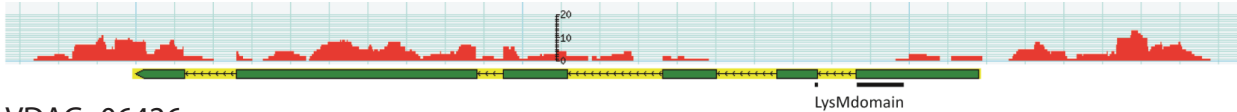

VDAG\_06426

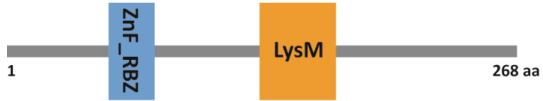

Supplement: Supplementary file 1 — Fig. S1 Disqualification of two previously identified Verticillium dahliae LysM effector genes. Two originally identified VdLysM effector genes are not predicted correctly. The initially predicted gene model of LysM effector gene VDAG_03096 is not supported by the mapping of RNA sequencing reads (in red). Reads map to predicted introns (in yellow), whereas some coding parts of the gene (in green), including the LysM domain that constitutes only a small part of the predicted protein, is not supported by reads. SMART prediction using the amino acid sequence encoded by VDAG_06426 reveals the absence of a signal peptide and the presence of a zinc finger domain. Also in this case, the LysM domain constitutes only a small portion of the predicted protein. [file MPP-18-596-s001.pdf]

Vd2LysM

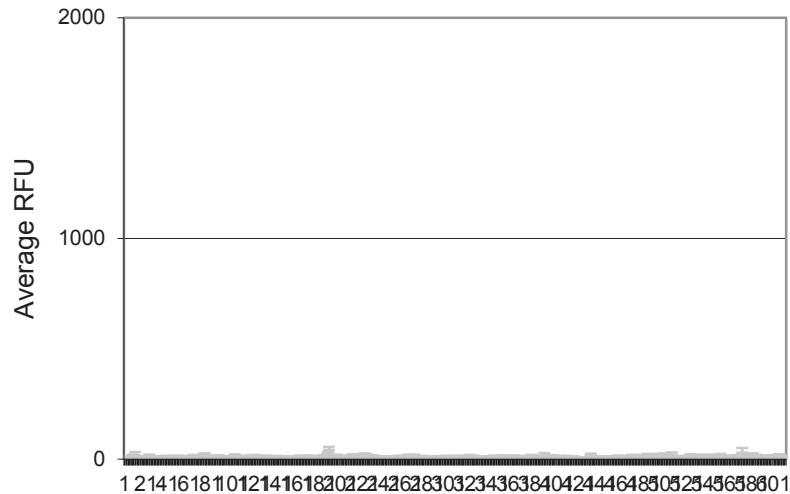

Ecp6

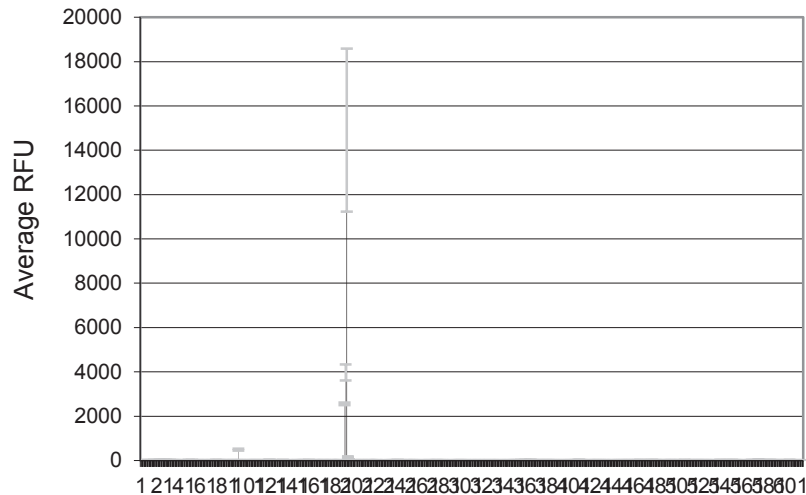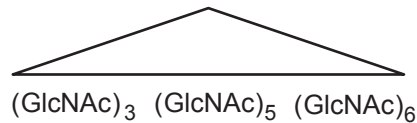

Supplement: Supplementary file 3 — Fig. S3 Glycan array analysis of Pichia pastoris‐produced Vd2LysM and Ecp6. Relative fluorescence (RFU, relative fluorescence unit) on scanning of a glycan array that contains probes for 406 glycans after hybridization with Vd2LysM and with Ecp6 as a control. Only Ecp6 hybridizes to the array, and only to 170–172, representing (GlcNAc)6, (GlcNAc)5 and (GlcNAc)3, respectively. [file MPP-18-596-s003.pdf]
