# Supplementary material for: Verticillium dahliae LysM effectors differentially contribute to virulence on plant hosts
Source: Mol Plant Pathol. 2017 Feb 14;18(4):596–608. doi: 10.1111/mpp.12520 (PMC6638240; doi:10.1111/mpp.12520)
Supplement: Supplementary file 4 — Table S1 Verticillium dahliae strains used in this study. [file MPP-18-596-s004.docx]

| Strain | Race | Originating host | Location |
| --- | --- | --- | --- |
| VdLs17 | 2 | Lettuce | Ca, USA |
| JR2 | 1 | Tomato | ON, Canada |
| CBS381.66 | 1 | Tomato | QC, Canada |
| St14.01 | 1 | Pistachio | CA, USA |
| St100 | 2 | Soil | Belgium |
| DVD-3 | 2 | Potato | Canada |
| DVD-31 | 2 | Tomato | Canada |
| DVD161 | 2 | Potato | ON,  Canada |
| DVD-S26 | 2 | Soil | Canada |
| DVD-S29 | 2 | Soil | Canada |
| DVD-S94 | 2 | Soil | Canada |
| JKG8 | 2 | Potato | The Netherlands |
| 2009-605 | 1 | Bell pepper | Ukraine |
| 463 | 2 | Cotton | Mexico |
| St16.01 | 2 | Cotton | Syria |
| V152 | 2 | Oak | Hungry |
| Vd52 | 1 | Pepper | Austria |
| Vd39 | 2 | Sunflower | Germany |
| Vd57 | 2 | Strawberry | Germany |
| van Dijk | 2 | Chrysanthemum | The Netherlands |

Supplemental Table 1. *Verticillium dahliae* strains used in this study
