# Supplementary material for: Verticillium dahliae LysM effectors differentially contribute to virulence on plant hosts
Source: Mol Plant Pathol. 2017 Feb 14;18(4):596–608. doi: 10.1111/mpp.12520 (PMC6638240; doi:10.1111/mpp.12520)
Supplement: Supplementary file 5 — Table S2 Primers used in this study. [file MPP-18-596-s005.docx]

| **Primer name** | **Primer sequence 5’-3’** | **For** |
| --- | --- | --- |
| Vd4LysM-RB_F | GGACTTAAUGTAGCTTTGGAGAGATGAGG | KO Vd4LysM |
| Vd4LysM-RB_R | GGGTTTAAUCGTAGATCTTCTGGAGAACC | KO Vd4LysM |
| Vd4LysM-LB_F | GGTCTTAAUTAGAGGTTGAAGGAGAGGAG | KO Vd4LysM |
| Vd4LysM-LB_R | GGCATTAAUTGTACTGGCAATGACAATCT | KO Vd4LysM |
| Vd5LysM-RB_F | GGACTTAAUGGAGTTGGAAAGGTCAGTAG | KO Vd5LysM |
| Vd5LysM-RB_R | GGGTTTAAUTCAATCAACAAGAGACAGGA | KO Vd5LysM |
| Vd5LysM-LB_F | GGTCTTAAUAGTTCATGAAGACGGAGAAG | KO Vd5LysM |
| Vd5LysM-LB_R | GGCATTAAUCATTCATGTCTAGCGAGGTA | KO Vd5LysM |
| Vd6LysM-RB_F | GGACTTAAUAGGAGCTGTAAGGACTGAAG | KO Vd6LysM |
| Vd6LysM-RB_R | GGGTTTAAUTCAAGATAAACCACGAGAGA | KO Vd6LysM |
| Vd6LysM-LB_F | GGTCTTAAUCATGACACGACAGATAGGAG | KO Vd6LysM |
| Vd6LysM-LB_R | GGCATTAAUTTTACATAATGGCGAGAGTG | KO Vd6LysM |
| Vd2LysM-RB_F | GGACTTAAUAGTTTTGCCTGACAGTAGGT | KO Vd2LysM |
| Vd2LysM-RB_R | GGGTTTAAUAATTGATAGTGAACGGCTTC | KO Vd2LysM |
| Vd2LysM-LB_F | GGTCTTAAUGGTTTTCTTACGCCAGTATC | KO Vd2LysM |
| Vd2LysM-LB_R | GGCATTAAUTTGTCTGACATGTTTCTCGT | KO Vd2LysM |
| Q-Vd2LysM-F | CCGAAGGACATGCAGTCATACCGG | Expression Vd2LysM |
| Q-Vd2LysM-R | TGCTGATATGGTTCCATTCCGTGAGG | Expression Vd2LysM |
| Q-VdGAPDH-F | CGAGTCCACTGGTGTCTTCA | Vd2LysM expression+ biomass VdLs17 |
| Q-VdGAPDH-R | CCCTCAACGATGGTGAACTT | Vd2LysM expression+ biomass VdLs17 |
| Q-Slrub-F | GAACAGTTTCTCACTGTTGAC | Biomass VdLs17 |
| Q-Slrub-R | CGTGAGAACCATAAGTCACC | Biomass VdLs17 |
| Vd2LysM-*Pich-*F | CGGTATGAATTCcatcatcatcatcatcatcccgactacaa..  ..ggacgacgatgacaagTACCGAAGGACATGCAGTCATAC | Vd2LysM *Pichia* production |
| Vd2LysM-*Pich-*R | cgtCTAGCGGCCGCTTAGTTCCAGCTGCACGGC | Vd2LysM *Pichia* production |
| Vd2LysM-plant-F | ACTAGTTACCGAAGGACATGCAGTCATAC | Vd2LysM *in planta* production |
| Vd2LysM-plant-R | GAGCTCTTAGTTCCAGCTGCACGGC | Vd2LysM *in planta* production |

Supplemental Table 2. Primers used in this study.
