# Supplementary material for: Verticillium dahliae LysM effectors differentially contribute to virulence on plant hosts
Source: Mol Plant Pathol. 2017 Feb 14;18(4):596–608. doi: 10.1111/mpp.12520 (PMC6638240; doi:10.1111/mpp.12520)
Supplement: Supplementary file 2 — Fig. S2 Sequence polymorphisms of core VdLysM effectors in the Verticillium dahliae population. Synonymous and non‐synonymous polymorphisms and their occurrence in 19 V. dahliae strains are indicated per VdLysM effector gene, and nucleotide and amino acid positions of polymorphisms are indicated, respectively. [file MPP-18-596-s002.pdf]

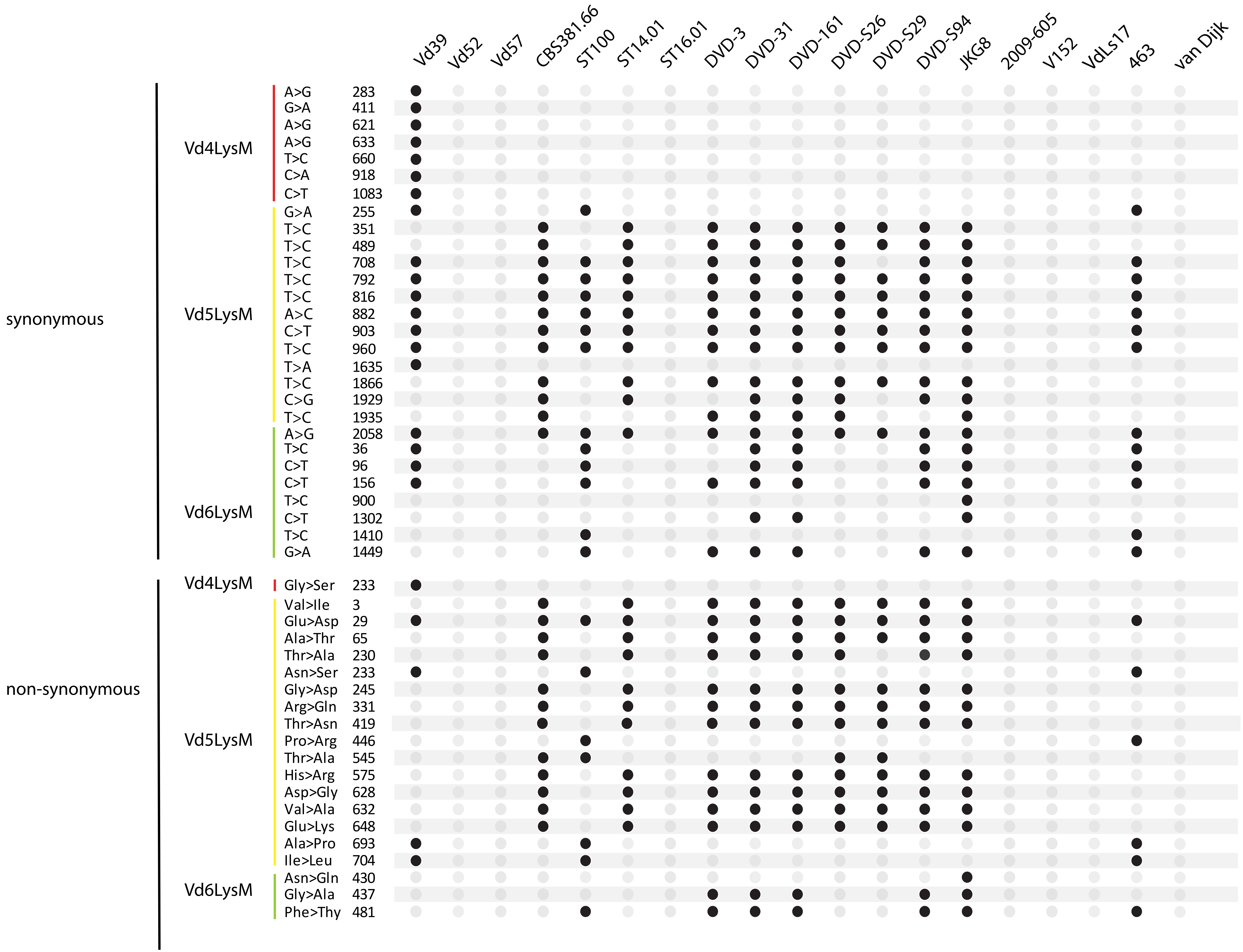
Vd39
Vd52
Vd57
CBS381.66
ST100
ST14.01
ST16.01
DVD-3
DVD-31
DVD-161
DVD-S26
DVD-S29
DVD-S94
JKG8
2009-605
V152
VdLs17
463
van Dijk
synonymous
Vd4LysM
A>G 283
G>A 411
A>G 621
A>G 633
T>C 660
C>A 918
C>T 1083
Vd5LysM
G>A 255
T>C 351
T>C 489
T>C 708
T>C 792
T>C 816
A>C 882
C>T 903
T>C 960
T>A 1635
T>C 1866
C>G 1929
T>C 1935
A>G 2058
Vd6LysM
T>C 36
C>T 96
C>T 156
T>C 900
C>T 1302
T>C 1410
G>A 1449
non-synonymous
Vd4LysM
Gly>Ser 233
Vd5LysM
Val>Ile 3
Glu>Asp 29
Ala>Thr 65
Thr>Ala 230
Asn>Ser 233
Gly>Asp 245
Arg>Gln 331
Thr>Asn 419
Pro>Arg 446
Thr>Ala 545
His>Arg 575
Asp>Gly 628
Val>Ala 632
Glu>Lys 648
Ala>Pro 693
Ile>Leu 704
Vd6LysM
Asn>Gln 430
Gly>Ala 437
Phe>Thy 481
